# Anatomical-guided third-generation laser balloon ablation for the treatment of paroxysmal atrial fibrillation assessed by continuous rhythm monitoring: results from a multicentre prospective study

**DOI:** 10.1093/europace/euae263

**Published:** 2024-11-07

**Authors:** Giuseppe Ciconte, Marco Schiavone, Giovanni Rovaris, Raffaele Salerno, Marzia Giaccardi, Elisabetta Montemerlo, Alessio Gasperetti, Elena Piazzi, Gabriele Negro, Stella Cartei, Roberto Rondine, Antonio Boccellino, Gianfranco Mitacchione, Mattia Pozzi, Mirko Casiraghi, Sergio De Ceglia, Roberto Arosio, Zarko Calovic, Gabriele Vicedomini, Giovanni B Forleo, Carlo Pappone

**Affiliations:** Faculty of Medicine and Surgery, Vita-Salute San Raffaele University, Via Olgettina 58, 20097 Milan, Italy; Arrhythmia and Electrophysiology Center, IRCCS Policlinico San Donato, Piazza E. Malan 1, 20097 San Donato Milanese, Milan, Italy; Department of Clinical Electrophysiology & Cardiac Pacing, Centro Cardiologico Monzino, IRCCS, Milan, Italy; Cardiology Unit, Fondazione IRCCS San Gerardo dei Tintori, Monza, Italy; Faculty of Medicine and Surgery, Vita-Salute San Raffaele University, Via Olgettina 58, 20097 Milan, Italy; Department of Cardiology, Ospedale Santa Maria Annunziata, Bagno a Ripoli, Florence, Italy; Meyer Children’s Hospital, Istituto di Ricovero e Cura a Carattere Scientifico (IRCCS), Florence, Italy; Cardiology Unit, Fondazione IRCCS San Gerardo dei Tintori, Monza, Italy; Department of Cardiology, ASST Fatebenefratelli-Sacco, Luigi Sacco University Hospital, Viale G.B. Grassi 74, 20157 Milan, Italy; Cardiology Unit, Fondazione IRCCS San Gerardo dei Tintori, Monza, Italy; Arrhythmia and Electrophysiology Center, IRCCS Policlinico San Donato, Piazza E. Malan 1, 20097 San Donato Milanese, Milan, Italy; Department of Cardiology, Ospedale Santa Maria Annunziata, Bagno a Ripoli, Florence, Italy; Arrhythmia and Electrophysiology Center, IRCCS Policlinico San Donato, Piazza E. Malan 1, 20097 San Donato Milanese, Milan, Italy; Faculty of Medicine and Surgery, Vita-Salute San Raffaele University, Via Olgettina 58, 20097 Milan, Italy; Arrhythmia and Electrophysiology Center, IRCCS Policlinico San Donato, Piazza E. Malan 1, 20097 San Donato Milanese, Milan, Italy; Department of Cardiology, ASST Fatebenefratelli-Sacco, Luigi Sacco University Hospital, Viale G.B. Grassi 74, 20157 Milan, Italy; Cardiology Unit, Fondazione IRCCS San Gerardo dei Tintori, Monza, Italy; Cardiology Unit, Fondazione IRCCS San Gerardo dei Tintori, Monza, Italy; Cardiology Unit, Fondazione IRCCS San Gerardo dei Tintori, Monza, Italy; Department of Cardiology, ASST Fatebenefratelli-Sacco, Luigi Sacco University Hospital, Viale G.B. Grassi 74, 20157 Milan, Italy; Arrhythmia and Electrophysiology Center, IRCCS Policlinico San Donato, Piazza E. Malan 1, 20097 San Donato Milanese, Milan, Italy; Arrhythmia and Electrophysiology Center, IRCCS Policlinico San Donato, Piazza E. Malan 1, 20097 San Donato Milanese, Milan, Italy; Department of Cardiology, ASST Fatebenefratelli-Sacco, Luigi Sacco University Hospital, Viale G.B. Grassi 74, 20157 Milan, Italy; Faculty of Medicine and Surgery, Vita-Salute San Raffaele University, Via Olgettina 58, 20097 Milan, Italy; Arrhythmia and Electrophysiology Center, IRCCS Policlinico San Donato, Piazza E. Malan 1, 20097 San Donato Milanese, Milan, Italy

**Keywords:** Atrial fibrillation, Laser balloon ablation, Pulmonary vein isolation, Continuous rhythm monitoring, Implantable cardiac monitors

## Abstract

**Aims:**

The third-generation laser balloon (LB3) is an established ablation device for pulmonary vein isolation (PVI) that allows direct visualization of the anatomical target. Equipped with an automatic circumferential laser delivery modality, it aims at continuous circumferential PVI, improving both acute and clinical outcomes. We sought to evaluate the clinical efficacy of LB3 ablation using an anatomical-based approach without verifying electrical isolation.

**Methods and results:**

Among 257 paroxysmal AF patients undergoing LB3 ablation across four Italian centres, 204 (72% male, mean age 60.4 ± 11.1 years) were included. The primary endpoint was freedom from any atrial tachyarrhythmia (ATa) recurrence after the blanking period (BP), assessed with implantable cardiac monitors (ICMs). All pulmonary veins (PVs) were targeted using the LB3, with the RAPID mode used on an average of 96 ± 8, 86 ± 19, 98 ± 11, and 84 ± 15% for the left superior, left inferior, right superior, right inferior PV, and left common ostium, respectively. Freedom from arrhythmia recurrences was 84.8% at 1, 80.4% at 2, and 76.0% at 3 years. An ATa burden ≥ 5% was documented in 2.5, 4.4, and 5.4% at 1, 2, and 3 years, respectively. Relapses during the BP [hazard ratio (HR) = 2.182, *P* = 0.032] and left atrial dilation (HR = 1.964, *P* = 0.048) were independent predictors of recurrences.

**Conclusion:**

Anatomical-guided LB3 ablation for paroxysmal AF is a safe and effective approach, providing excellent clinical outcomes as assessed by ICM over nearly 3 years of follow-up.

## Graphical Abstract

**Graphical Abstract:**
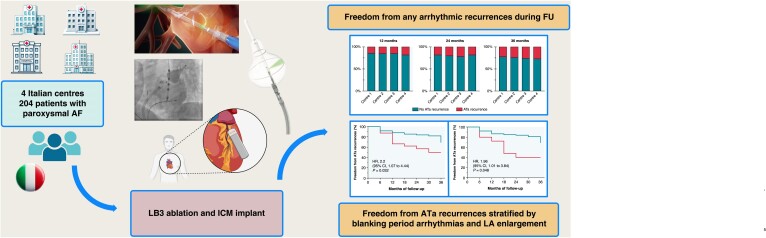

**What’s new?**

- This is the first multicentre study reporting the clinical outcome of third-generation laser balloon ablation for paroxysmal atrial fibrillation using an anatomical-based strategy without routine verification of post-ablation electrical isolation.
- Freedom from atrial tachyarrhythmia recurrences assessed by continuous rhythm monitoring using implantable cardiac monitors is 76% over nearly 3 years of follow-up using this approach.
- Anatomical pulmonary vein isolation can be achieved using an automatic modality of laser delivery (RAPID mode) in more than 90% of targeted veins.
- Moderate left atrial dilation and arrhythmic events during the blanking period significantly predict post-ablation atrial tachyarrhythmia recurrences, providing valuable insights for patient selection and management.

## Introduction

Catheter ablation aiming at electrical isolation of the pulmonary veins (PVs) has become a cornerstone treatment for managing paroxysmal atrial fibrillation (PAF).^1^ However, achieving highly reproducible isolation remains challenging due to ineffective lesions caused by lack of transmurality and electrical gaps, which can lead to post-ablation recurrence of atrial fibrillation (AF) or atrial tachyarrhythmias (ATas).^2^ Balloon-based ablation techniques have been designed to offer a solution to create contiguous lesions that are less dependent on operator dexterity.^3^ Several catheter-based balloon devices utilizing various energy sources are available to enhance the efficacy and safety of the ablation procedure. Among these systems, the visually guided laser balloon has emerged as a promising technique, offering clinical outcomes comparable to those of radiofrequency (RF) and cryoballoon (CB) pulmonary vein isolation (PVI).^4,5^ By allowing direct visualization of the PV antrum, it allows laser energy delivery to achieve circumferential ablation.^6^ Recent innovations have introduced the third-generation laser balloon (LB3; X3, HeartLight, CardioFocus, Marlborough, MA, USA) with technical advancements aimed at improving ablation outcomes. This novel catheter has been equipped with an integrated motor to automate circumferential lesion creation (RAPID mode™), potentially increasing the speed and efficacy of the procedure. This technique delivers contiguous laser energy around the PV antrum with real-time visualization, ensuring precise and effective lesion formation. Preliminary results indicate shorter procedure times with less fluoroscopy and a high rate of first-pass electrical isolation, demonstrating higher clinical efficacy compared to the second-generation balloon. The latter shows similar outcomes to RF and CB PVI techniques.^4,5,7,8^ Early clinical experiences demonstrated a significant rate of acute PVI with the LB3, suggesting that routine post-ablation verification of electrical isolation might be unnecessary when dealing with this energy source if a circumferential anatomical-based ablation approach is completed. Therefore, this multicentre study aimed to evaluate the feasibility, safety, and clinical efficacy of LB3 ablation using an anatomical-based ablation strategy.

## Methods

This study is a multicentre prospective study that includes patients undergoing LB3 ablation for the treatment of PAF. The study was conducted in accordance with the principles of the Declaration of Helsinki. The study was approved by the local institutional ethics committee, and all patients provided written informed consent.

### Study population

Since September 2019, all patients with drug-refractory PAF^1^ undergoing LB3 ablation at four electrophysiology centres in Italy (Centre 1, Centre 2, Centre 3, and Centre 4) were included in this registry. Patients with less than 6 months of follow-up were not included in the outcome analysis. Exclusion criteria included age < 18 years, persistent AF, prior AF ablation procedure, hyperthyroidism, severe left atrial enlargement, intracardiac thrombi, severe valvular heart disease, uncontrolled heart failure, contraindications to oral anticoagulant (OAC) therapy, myocardial infarction or unstable angina, recent coronary bypass (<6 months), pregnancy, active infectious disease, and significant comorbidities such as cancer, severe renal failure requiring dialysis, severe obstructive pulmonary disease, and cirrhosis, with a life expectancy of less than 2 years.

### Pre-procedural management

Pre-procedural transoesophageal echocardiography was performed to exclude intracardiac thrombi and to assess atrial morphology in all patients. An uninterrupted anticoagulation scheme with direct anticoagulants or with warfarin was used.

### Ablation procedure

All procedures were performed under general anaesthesia (GA) or deep sedation (DS) using midazolam, fentanyl, propofol, and/or dexmedetomidine. According to operators’ preferences, an oesophageal probe was used to monitor oesophageal temperature during ablation procedures. The cut-off temperature was set at 40°C: if oesophageal temperature went above the cut-off value, energy delivery was interrupted and ablation was continued using reduced energy or at different locations (i.e. more distal or more proximal). After obtaining left atrium (LA) access via a single transseptal puncture, a 12 Fr steerable sheath was advanced into the LA. Following the transseptal puncture, heparin boluses were administered to maintain an activated clotting time ≥ 300 s throughout the procedure, as also previously reported.^6^ No mapping catheter was introduced to assess PV potentials or to obtain a complete electroanatomical map of the LA. Pulmonary vein anatomy was assessed by pre-procedural transoesophageal echocardiography or by selective angiography prior to ablation. The balloon was inflated by the operators using the controller located next to the catheter handle. Tissue contact with the balloon was observed exclusively through the endoscopic view. The standard laser output power was set at 13–15 W in RAPID mode when stable contact was achieved without blood flow on the balloon surface. Catheter manipulations or rotations were always applied to improve tissue contact and to better expose the target area. This approach was aimed to adopt the RAPID mode over the point-by-point ablation modality, which was applied (8.5 W for 20 s or 5.5 W for 30 s) where blood flow or pooling was close to the target tissue due to poor contact. During ablation of the right-sided PVs, a decapolar catheter was placed in the superior vena cava to assess phrenic nerve capture. After having completed circumferential PV lesions, the laser balloon was then removed, and post-procedural PVI was not assessed.

### Post-procedural care and continuous monitoring

The presence of pericardial effusion was checked with transthoracic echocardiography at the end of the procedure. Direct anticoagulants or warfarin was resumed in the early postoperative period and continued for at least 3 months. Antiarrhythmic drug therapy was introduced after ablation and/or continued during the blanking period and generally discontinued after 3 months at the physician’s discretion. To evaluate ATa recurrences, continuous monitoring was implemented for all patients. This included the use of an insertable cardiac monitor (ICM) for patients without prior devices, ensuring the detection and follow-up of AF recurrences within a 1–2-month peri-procedural period. Patients were followed with clinical visits and a 12-lead electrocardiogram at 3-, 6-, and 12-month post-procedure and subsequently at the discretion of the treating cardiologist. Automatic transmissions from the ICM were obtained periodically or in the event of an arrhythmic event recording.

### Data collection and study outcomes

Demographic, medical history, procedural, peri-procedural, and follow-up data were collected into a centralized anonymized database. All identified arrhythmic events detected by the ICMs were first evaluated by at least one blinded and independent electrophysiologist at each centre. The primary endpoint was the first recurrence of AF identified by the ICMs, 90 days after the procedure (blanking period). Freedom from AF recurrences was assessed at 3-, 6-, and 12-month follow-up and subsequently every 6 months or at the discretion of the treating cardiologist. Data on AF burden were collected at each clinical evaluation, defined as the cumulative duration of all AF episodes lasting ≥6 min from the first adjudicated AF episode onward, divided by the total duration of monitoring. All ATa recurrences detected by the ICMs were categorized into any ATa episode. Data on discontinuation of antiarrhythmic and anticoagulant drugs were also collected.

### Statistical analysis

Continuous variables are expressed as mean ± standard deviation and were compared using one-way ANOVA or independent *t*-tests for pairwise comparisons between centres. Categorical variables, presented as absolute numbers and percentages, were compared using the *χ*^2^ test or Fisher’s exact test, with pairwise comparisons between centres using Fisher’s exact test. Event-free survival was estimated using the Kaplan–Meier method, and survival curves were compared using the log-rank test. Cox proportional hazards regression models were used to identify predictors of arrhythmic recurrences through univariate and multivariate analyses. Stepwise regression based on likelihood ratios was applied for multivariate models, with hazard ratios (HRs) reported alongside 95% confidence intervals (CIs) and *P* values from the Wald test. Statistical significance was set at *P* < 0.05. Statistical analyses were conducted using GraphPad Prism (GraphPad Software, San Diego, CA).

## Results

### Baseline characteristics

Between September 2019 and May 2024, a total of 257 patients underwent ablation. Thirty-four patients were excluded from the analysis due to refusal of ICM implantation. An additional 19 patients were not considered as they had less than 6 months of follow-up. A total of 204 patients (72% male, mean age 60.4 ± 11.1 years) with PAF underwent LB3 catheter ablation across four Italian centres. The mean interval from the initial AF episode to ablation was 55.7 ± 66.3 months, with an average of 5 ± 3 AF-related visits to the emergency department or clinic per patient. The mean left ventricular ejection fraction was 59.2 ± 5.0%, with 16% (*n* = 33) presenting with moderate mitral regurgitation. Additionally, the mean left atrial volume/body surface area was 31.4 ± 7.2 mL/m², with 7% of patients (*n* = 15) presenting with moderate left atrial dilation. All patients had undergone at least one trial of antiarrhythmic drug therapy prior to the procedure, and the majority of patients, 79% (*n* = 161), were on chronic OAC therapy. Baseline characteristics of the patients are detailed in *Table 1*.

**Table 1:** Baseline and clinical characteristics of the study population

	Total (*n* = 204)
Patients, *n* (%)	
Centre 1	65 (32)
Centre 2	71 (35)
Centre 3	46 (23)
Centre 4	22 (11)
Age (years)	60.4 ± 11.1
Male, *n* (%)	146 (72)
BMI (kg/m^2^)	26.2 ± 4.3
Creatinine (mg/dL)	0.94 ± 0.24
Comorbidities, *n* (%)	
Heart failure	9 (4)
CAD	17 (8)
Previous TIA/stroke	8 (4)
Hypertension	117 (57)
Dyslipidaemia	92 (45)
Chronic obstructive pulmonary disease	3 (1)
Diabetes	17 (8)
OSAS	12 (6)
Echo findings	
LVEF (%)	59.2 ± 5.0
LA volume/BSA (mL/mq)	31.4 ± 7.2
Moderate LA enlargement, *n* (%)	15 (7)
Moderate MI, *n* (%)	33 (16)
Drug therapy, *n* (%)	
Oral anticoagulation	161 (79)
Antiaggregant	13 (6)
β-Blockers	114 (56)
ACE-I/ARB/ARNI	87 (43)
ARA	8 (4)
SGLT2I	2 (1)
Statins	41 20)
CCB	7 (3)
Time from first AF episode (months)	55.7 ± 66.3
ER/clinic visits AF-related, *n* (mean ± SD)	4.8 ± 2.8

Data are reported as mean ± standard deviation or number (percentage) in case of binary variables.

ACE-I, angiotensin-converting enzyme inhibitors; AF, atrial fibrillation; ARB, angiotensin II receptor blockers; ARA, aldosterone receptor antagonists; ARNI, angiotensin receptor-neprilysin inhibitors; BMI, body mass index; BSA, body surface area; CAD, coronary artery disease; CCB, calcium channel blockers; ER, emergency room; LA, left atrium; LVEF, left ventricle ejection fraction; MI, mitral insufficiency; OSAS, obstructive sleep apnoea syndrome; SGLT2I, sodium-glucose co-transporter 2 inhibitors; TIA, transient ischaemic attack.

### Procedural characteristics

**Figure 1:**
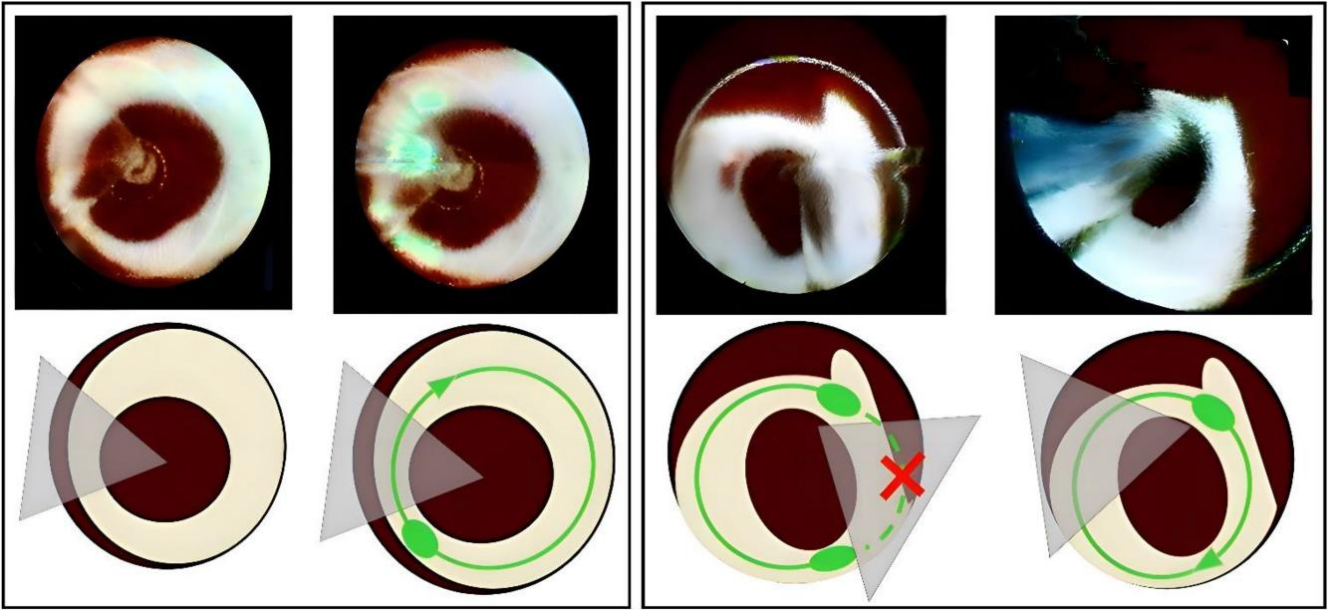
This figure demonstrates the LB ablation technique with rapid modality for circumferential PVI, comparing different approaches based on varying levels of contact with the PV antrum. (*Left panels*) The target is the RSPV. Top panels: the LB correctly occludes the RSPV, with the blind spot free, making single-sweep ablation feasible. The laser beam navigates through the blind spot, allowing for complete circumferential isolation. Bottom diagrams: schematic representation of the laser ablation process for the RSPV. The accurate evaluation confirms the laser beam’s entry and exit from the blind spot, ensuring successful circumferential isolation (contiguous circumferential laser delivery around the PV antrum using RAPID mode; solid green line). (*Right panels*) The target is the LIPV. Top panels: initial assessment of LB occlusion shows poor catheter-to-vein coaxiality due to the vein's anatomy, creating challenges from potential blood contact with the laser beam. Balloon rotation exposes the target area, enabling a two-step RAPID mode laser delivery for a complete circumferential lesion. Bottom diagrams: the double-step RAPID mode is employed to achieve complete circumferential isolation. The solid green line indicates laser energy delivery in RAPID mode, while the dashed line represents the projection of the laser beam not delivered due to missing tissue contact. This figure highlights the need for differential strategies in LB ablation depending on the anatomical alignment of the PVs, ensuring effective and safe ablation for PV isolation.

The procedural and peri-procedural data are presented in *Table 2*. General anaesthesia was used in 54% (*n* = 110) of cases, while the remaining procedures were performed under DS. All PVs were circumferentially targeted with the LB3, and no immediate post-ablation verification of electrical isolation was performed (*Figure 1*). The mean total procedure time was 52.3 ± 16.0 min, with a mean fluoroscopy time of 5.3 ± 2.5 min. Overall, there were few significant differences across centres, such as age, dyslipidaemia, procedure, and fluoroscopy times (*P* = 0.004, *P* = 0.037, *P* < 0.001, and *P* = 0.002, respectively; Supplementary material online, *Table S1*). However, these variations did not impact clinical outcomes (see Supplementary material online, *Table S1*). Anatomical variants were found in 19% of patients, with 33 left common ostia, 2 intermediate branches, and 3 right common ostia. Eighty-eight per cent of patients were in sinus rhythm at the beginning of the procedure. The mean laser time was 5.0 ± 3.0, 5.0 ± 2.1, 4.4 ± 2.0, 4.6 ± 2.1, and 5.4 ± 1.7 min for the left superior pulmonary veins (LSPVs), left inferior pulmonary veins (LIPVs), right superior pulmonary veins (RSPVs), right inferior pulmonary veins (RIPVs), and left common ostium (LC), respectively. The average use of the RAPID mode on the percentage of vein circumference was 96 ± 8, 86 ± 19, 98 ± 11, 88, and 84 ± 15% for the LSPV, LIPV, RSPV, RIPV, and LC, respectively. In this cohort, 110 patients were managed with GA, while 94 patients received DS (see Supplementary material online, *Table S2*). There were no significant differences in clinical characteristics or freedom from arrhythmia recurrences following catheter ablation. However, patients under DS had a higher prevalence of hypertension and experienced longer procedure and fluoroscopy times (see Supplementary material online, *Table S2*). Oesophageal monitoring was utilized in 81 patients (39.7%) based on physician preference. In 13 of these patients (16%), an increase in oesophageal temperature necessitated the interruption of energy delivery. Laser delivery was subsequently resumed at a more distal or proximal location relative to the area where the temperature rise occurred. In only 4% of these cases, the balloon was repositioned to target a different area to prevent further temperature elevation. There were no peri-procedural deaths. Two patients (2/204; 0.9%) experienced pericardial effusion, only one of them (1/204; 0.4%) requiring pericardiocentesis. There were three (3/204; 1.5%) cases of phrenic nerve palsy: two (2/204; 1.5%) of them transient resolving during the follow-up and only one (1/204; 0.4%) persistent at the latest follow-up. Other complications included two access site complications, one of them required surgical treatment. None of the patients experienced an acute thromboembolic event (stroke or transient ischaemic attack) or atrioesophageal fistulas. In one case, a balloon rupture was documented after left PV ablation requiring exchange of a new LB.

**Table 2:** Procedural and peri-procedural data

	Total (*n* = 204)
Total procedure time (min)	52.3 ± 16.0
Fluoroscopy time (min)	5.3 ± 2.5
General anaesthesia, *n* (%)	110 (54)
Deep sedation, *n* (%)	94 (46)
Baseline sinus rhythm, *n* (%)	180 (88)
Anatomical variant, *n* (%)	38 (19)
Left common ostia	33 (16)
Right common ostia	3 (1)
Intermediate branches	2 (1)
% RAPID mode use	
LSPV	96 ± 8
LIPV	86 ± 19
RSPV	98 ± 11
RIPV	88 ± 27
Left common ostia	84 ± 15
Laser ablation time (min)	
LSPV	5.0 ± 3.0
LIPV	5.0 ± 2.1
RSPV	4.4 ± 2.0
RIPV	4.6 ± 2.1
Left common ostia	5.4 ± 1.7
Procedural complications, *n* (%)	
Deaths	0 (0)
Peri-procedural stroke/TIA	0 (0)
Pericardial effusion	2 (0.9)
Persistent Phrenic nerve palsy	1 (0.5)
Vascular access site complications	2 (0.9)
Balloon rupture	1 (0.5)

Data are reported as mean ± standard deviation or number (percentage) in case of binary variable.

LIPV, left inferior pulmonary vein; LSPV, left superior pulmonary vein; RIPV, right inferior pulmonary vein; RSPV, right superior pulmonary vein; TIA, transient ischaemic attack.

### Follow-up and clinical outcomes

**Figure 2:**
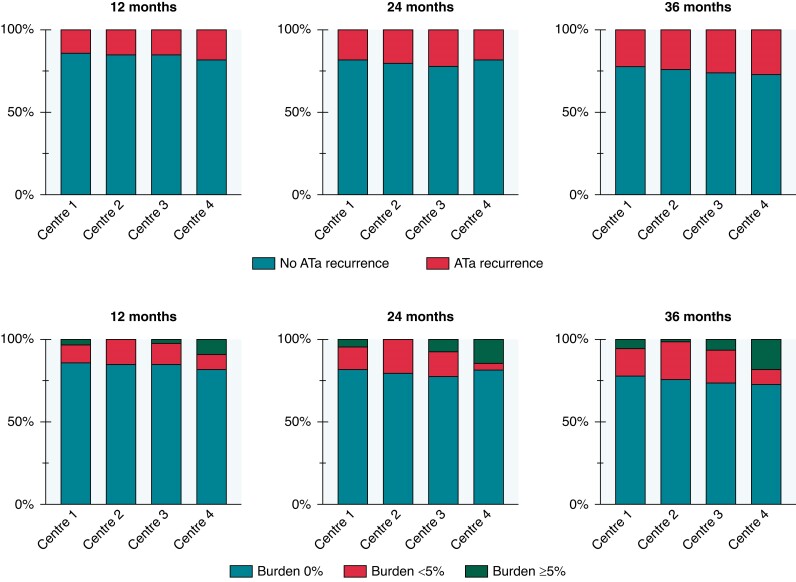
Proportion of patients with and without any atrial tachyarrhythmias (ATa) at implanted device interrogation and different arrhythmic burden cut-off (0, <5, and ≥5%) at 12-, 24-, and 36-month follow-up in the four centres.

**Figure 3:**
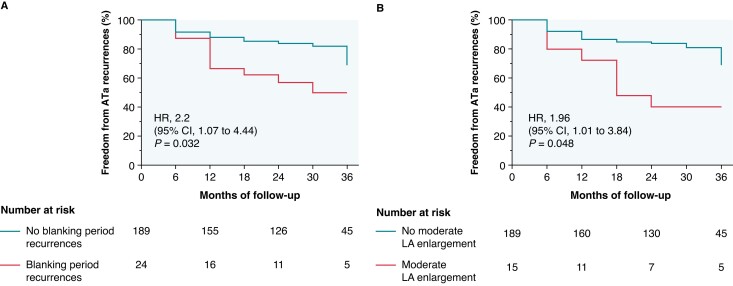
Time to first atrial tachyarrhythmias (ATa) episode. Kaplan–Meier survival curves for any ATa recurrences after a 3-month blanking period in patients with or without variables significantly associated with an increased risk of arrhythmic recurrence. (*A*) Different survival curves between patients with or without arrhythmic recurrences during the blanking period. (*B*) Different survival curves between patients with or without moderate left atrium (LA) enlargement.

The mean follow-up period was 34.9 ± 9.8 months. Freedom from any ATa recurrences after the blanking period was 84.8% at 1 year, 80.4% at 2 years, and 76.0% at 3 years (*Figure 2*). Atrial tachyarrhythmia recurrence rate with burden ≥ 5% was 2.5% at 1 year, 4.4% at 2 years, and 5.4% at 3 years (*Figure 2*). No significant differences were found among the four centres regarding arrhythmic recurrences (*P* = 0.93) (*Figure 2*). In 12% (*n* = 24) of patients, arrhythmic recurrences were documented during the blanking period, and in 46% of these (*n* = 11), arrhythmic recurrences were also recorded during the subsequent follow-up. Notably, 53% (*n* = 8) of patients with LA enlargement experienced arrhythmic recurrences during follow-up. After a single procedure, recordings of ATa during the blanking period (HR = 2.182, *P* = 0.032), as well as moderate atrial enlargement (HR = 1.964, *P* = 0.048), were found to be significantly associated with an increased risk of arrhythmic recurrence (*Table 3*). *Figure 3* presents the Kaplan–Meier curves for variables associated with an increased risk of arrhythmic recurrence. During follow-up, 10 patients underwent a redo procedure: 4 patients showed PV reconnections and 6 experienced isthmus-dependent right atrial flutter (during this procedure, PVs were checked and found to be still isolated). No left atrial flutter was documented. At the end of follow-up, 39% (*n* = 80) of patients discontinued anticoagulant therapy and 64% discontinued antiarrhythmic drugs, whereas 17% (*n* = 35) reduced the dose. During the follow-up period, three patients (1.4%) progressed to persistent AF. All presented with cardiovascular comorbidities: two had obesity, two had dyslipidaemia, one had diabetes, and all three had arterial hypertension. Their indexed left atrial volumes were 40.1, 41.6, and 43.5 mL/m², respectively.

**Table 3:** Proportional hazard cox models for the association of clinical variables with ATa recurrences after laser balloon ablation

	ATa episode					
	Univariate			Multivariate		
Variable	Hazard ratio	95% CI	*P* value	Hazard ratio	95% CI	*P* value
Age	1.022	[0.991, 1.054]	0.155	1.020	[0.986, 1.055]	0.242
Sex	1.008	[0.499, 2.037]	0.983	1.006	[0.497, 2.036]	0.987
BMI ≥ 25	0.740	[0.261, 2.100]	0.572	0.746	[0.256, 2.176]	0.598
Hypertension	0.631	[0.267, 1.491]	0.296	0.610	[0.253, 1.474]	0.271
CAD	0.891	[0.362, 2.191]	0.800	0.904	[0.362, 2.259]	0.828
Moderate LA enlargement	1.970	[1.019, 3.807]	0.043	1.964	[1.006, 3.838]	0.048*
Baseline sinus rhythm	1.039	[0.503, 2.147]	0.920	1.034	[0.498, 2.149]	0.928
Blanking period recurrence	2.177	[1.086, 4.365]	0.029	2.182	[1.073, 4.441]	0.032*

Significant values (*P* < 0.05) are marked with an asterisk (*).

ATa, atrial tachyarrhythmia; BMI, body mass index; CAD, coronary artery disease; CI, confidence interval; LA, left atrium.

## Discussion

This study focused on the long-term clinical outcomes of patients undergoing PAF ablation with the LB3. By analysing recurrence rates and maintenance of sinus rhythm without routine post-ablation verification of electrical isolation, this study seeks to provide insights into the reliability and clinical utility of an anatomical-based ablation strategy for PAF. The main findings of this study are the following: (i) the LB3 catheter ablation system allowed a safe and effective acute PVI; (ii) 3-year freedom from any ATa episode using ICM recordings was 76% at approximately 3 years of follow-up; and (iii) patients experiencing recurrences had a low arrhythmic burden and were essentially asymptomatic 3-year after the procedure. The evolution of catheter ablation techniques for PAF has been mostly driven by the need to improve procedural safety, efficacy, and patient outcomes, as well as overall procedural times. Pulmonary vein isolation remains fundamental in AF treatment, with ongoing efforts to enhance its effectiveness through advanced ablation technologies.^9^ Recent innovations, including balloon-based systems and pulsed field ablation (PFA), have improved the consistency and durability of PVI, especially with a focus on anatomical-guided approaches.^10^ Our study highlights the efficacy of the LB3 in achieving effective long-term arrhythmia freedom, aligning with the evolving landscape of AF ablation where new technologies are continuously being evaluated to improve outcomes.^10,11^ Balloon-based ablation and ‘one-shot’ technologies have been developed to address these limitations by providing a more straightforward and reproducible approach to PVI.^3^ The laser balloon catheter allows direct inner ‘surgical’ visualization of the PV anatomy to deliver precisely the energy source. Thus, unlike conventional methods that rely on electrophysiological mapping, this catheter facilitates an anatomical-based strategy by a purely visually guided circular ablation. Traditionally, LB ablation is carried out ‘blindly’ during the energy application, and PVI can be checked subsequently with a multipolar mapping catheter placed in the PV ostium after the completion of the ablation procedure. Previous clinical studies have highlighted the success rates of the second-generation LB ablation, with acute isolation rates and long-term safety and effective profile that are comparable to the ones achieved with other established ablation outcomes.^4,5,7,8^ However, LB ablation typically results in longer procedural times compared to CB PVI, as demonstrated by Chun *et al*.^4^ Nevertheless, later comparisons with others including LB3 patients, although accounting only for a small proportion of the entire cohort (12.7%), showed that procedural times were similar between the two techniques, with median overall procedural times of 87 [73–104] minutes for the LB.^5^ As observed in this study enrolling only patients undergoing LB3 ablation, this catheter allows for significantly reduced procedural times, up to a median of 52 ± 16 min. These results are corroborated by the absence of left atrial remapping and the extensive use of the RAPID mode in our cohort, which allows for reduced procedural times compared to previous LB generation. Remarkably, overall procedural times align with the ones observed in the latest registries using PFA technology, such as the MANIFEST-PF (61 min [range, 15–362]), the ATHENA (63 min, IQR [55–80]) and the EU-PORIA (58 min [IQR, range: 40–87]) projects.^12–14^ Only a minority of patients in these studies underwent more extended ablations than PVI only (22.8, 24, and 14%), thereby justifying slightly longer procedural PFA times compared to other results. From a technical point of view, the LB3 catheter integrates advanced features that enhance its efficacy and safety profile. The ability to visually confirm lesion formation in real time and the uniformity of the laser-induced lesions contribute to some potential advantages compared to other traditional ablation methods. The balloon design has been further modified to be continuously and dynamically adjustable, enhancing contact and optimizing the level of isolation, thus offering a versatile tool to achieve adequate PV occlusion and tissue contact also when dealing with irregular anatomies. The most remarkable innovation is characterized by the automated RAPID mode. During this ablation modality, the lesion generator is continuously swept around the PV ostium (either clockwise or counterclockwise) at a preset speed (2.25° per second) by an integrated motor. In comparison to the predecessor balloon, first-pass isolation rates further improved with the new ablation mode (89% vs. 95%).^15^ Since the mechanical balloon characteristics have not been changed, this is interpreted as a merit of continuous rather than point-by-point ablation leading to fewer acute conduction gaps in the lesion set. Moreover, the LB3 offers a high-power short-duration ablation with continuous energy delivery, as well as faster inflation and deflation of the LB. This technique offers several potential advantages, including the ability to visualize the ablation process in real time and create uniform lesion sets. In our clinical practice, following the initial successful experiences using the second-generation laser balloon in achieving a very high rate of acute successful PVI, and also supported by the clinical evidence achieving single-sweep PVI with the LB3, we started to investigate the efficacy of this procedure without routine verification of post-ablation PV electrical disconnection. This approach was based on the premise that the anatomical completeness of the lesions would correlate with sustained clinical efficacy. However, the very long-term clinical efficacy of this approach, particularly in the absence of immediate post-ablation electrophysiological confirmation, remains an area of investigation. As proven by continuous monitoring using ICM, in the present study, the success rate of the procedure was 76% as it was maintained at 3 years of follow-up, indicating the high efficacy of this approach. Considering the proof-of-concept approach adopted in this study, we only focused on patients carriers with ICM to provide a reliable method of arrhythmia detection.^16,17^ Remarkably, success rates obtained with the LB3 were significantly higher than those obtained with its predecessor, also with follow-up based on a similar monitoring strategy.^6^ These excellent clinical outcomes might be reflected by the improved catheter design which allows a better lesion contiguity provided by the automatic mode, as also reflected by the high rate of RAPID mode use in this study, thus minimizing the risk of electrical gaps. This observation is supported by the experience of Tohoku *et al*.^15^ reporting acute PVI rates > 90% associated with a high > 90% RAPID mode use per vein. Moreover, an anatomical-based approach may offer the advantage of reducing the overall cost of the procedure avoiding the use of multielectrode catheters to remap the PVs and of the use of the 3-D mapping system.^18^ In these historical times in which cost containment is of pivotal importance in the economics of the healthcare system, this advantage should not be underestimated. In fact, one could ultimately decide to check PVI only when a full circumferential LB ablation could not be satisfactorily achieved. In fact, the inability to visually control the ablation due to poor catheter-to-vein coaxiality and eccentric balloon positioning, or excessive interference of the blood in catheter to tissue contact, may affect the laser delivery to the tissue and may cause incomplete ablation or even complications (e.g. thrombus formation and/or balloon pinholes).^19^ The choice between GA and DS for catheter ablation has been a topic of debate, with implications for procedural efficiency and patient outcomes. In our study, patients treated under GA exhibited shorter fluoroscopy times, possibly reflecting more precise catheter manipulation and reduced radiation exposure, in agreement with previous experiences.^20,21^ These findings suggest that GA may offer several procedural advantages, potentially enhancing safety, and reducing procedure time and radiation exposure compared to the DS approach. However, both groups demonstrated similar long-term outcomes regarding arrhythmia recurrence, indicating that the choice of anaesthesia does not significantly impact the efficacy of the ablation procedure, as occurred in this series. Similarly, the higher prevalence of hypertension in the DS group did not lead to differences in procedural success or complication rates. Notably, only few significant differences across centres were observed, mostly regarding procedural aspects, with higher-volume centres showing shorter procedure and fluoroscopy times, likely due to greater experience and a steeper learning curve. Importantly, these variations did not affect clinical outcomes, and no significant differences were observed in the number of patients with AF burden > 5%. The slightly higher AF burden in one centre may reflect a learning curve effect in the lower-volume centre, further underscoring the impact of operator experience on procedural efficiency. In the current era of emerging technologies like PFA, cost considerations remain paramount. In fact, although PFA theoretically carries a lower risk of complications due to its myocardial selectivity, it is significantly expensive.^22^ While a systematic cost-effectiveness analysis of LB3 vs. PFA is beyond our study’s scope, the choice of catheter should consider both efficacy and cost. This is particularly relevant given the outcomes of our study, which demonstrate a high rate of freedom from arrhythmia recurrences and a low number of peri-procedural complications, unrelated to the energy source. However, one must acknowledge that the use of multielectrode mapping catheters integrated in a 3-D mapping system is a significant leap forward in terms of PV mapping catheter ablation, as it is for PFA lesion validation using non-pentaspline catheters. The latter offers the obvious advantage of assessing the electroanatomical mapping and eventual more versatile deployment of the lesion using focal catheters.^23^ As evident from our results, the benefit of using multipolar catheters is potentially outweighed by the higher risks of peri-procedural strokes due to catheter exchanges over the larger balloon sheath. Nevertheless, this study may open the future to a number of dosing studies in terms of laser application that will help us define the balance between ablating ‘too much’ and ‘not enough’. Larger studies are needed to determine whether LB ablation with the third-generation device can be performed by the sole guidance of complete PV occlusion without the need of verification of PVI. If these promising results would be confirmed, a pure anatomical-guided LB ablation would further simplify the ablation procedure and make it more cost-effective. Regarding efficacy outcomes, the use of a 5% burden cut-off to identify meaningful recurrences was derived from previous studies on the topic^24,25^ which identified these recurrences as the most significantly associated with cardiovascular hospitalization, ischaemic stroke, as well as deterioration of LV function. Moreover, longer recurrences have been associated with an increased thromboembolic risk.^26,27^ However, although offering the clinician enhanced granularity in rhythm monitoring, several important questions remain unanswered on the real clinical impact and the need for oral anticoagulation in the long term,^28^ especially regarding proper time cut-offs. In a more recent case-crossover trial,^29^ patients with a subclinical duration exceeding 5.5 h had a 3.7-fold increase in stroke risk, which escalated to a 5-fold increase for episodes exceeding 24 h. Other evidence points towards the highest risk pertaining to patients with the highest arrhythmia burden. Finally, no serious adverse events were observed in this study.^30,31^ In previous experiences, the phrenic nerve injury rate using LB ablation was reported to be in the range of 1.4–2%,^4,6^ while just one persistent phrenic nerve palsy (0.5%) occurred with the LB3 in our cohort. The shorter energy delivery times needed to isolate PV areas adjacent to the phrenic nerve with the RAPID mode is a potential explanation for our findings. This should be highlighted since the most important difference between LB and PFA so far is the absence of phrenic nerve injury detected in trials comparing these two techniques.^32,33^

### Limitations

Our study has some limitations. The non-randomized study design and the lack of a control group for comparison might limit the applicability of these results to other patient’s populations. The four centres are considered as mid- and high-volume EP laboratories, and all of them are currently using the same approach when dealing with LB ablation as demonstrated by homogenous overall freedom from arrhythmic recurrences. However, we acknowledge that variations in centre volumes could introduce a potential bias. The relatively higher number of patients with an AF burden > 5% recorded in one centre may be influenced by the learning curve associated with lower procedural volume compared to the other centres. Roughly 20% of patients had an ICM implanted well in advance of the procedure; thus, a pre-procedural AF burden estimation could not be systematically assessed. We did not systematically monitor all possible procedural complications (e.g. heart computed tomography scan for PV stenosis or oesophagogastroduodenoscopy to assess potential oesophageal lesions), and thus, the complication rate may have been underestimated. Furthermore, post-ablation endoscopy was not conducted, which may have prevented the detection of any subclinical oesophageal injury. However, no clinically relevant gastroesophageal complications were observed during the follow-up period. Future prospective randomized studies with larger samples and eventual comparison with other techniques (e.g. CB ablation or PFA) and same follow-up monitoring with ICMs might be needed to eventually show any difference in outcome between both methods (post-procedural isolation verification vs. not).

## Conclusions

Anatomical-guided LB3 ablation in the treatment of PAF is a safe and very effective approach affording excellent clinical outcome with high rates of freedom from arrhythmic recurrences as assessed by continuous rhythm monitoring after nearly 3 years of follow-up. Further randomized studies are desirable to finally confirm these promising results.

## Supplementary Material

euae263_Supplementary_Data

## Data Availability

The data underlying this article will be shared on reasonable request to the corresponding authors.
