# Supplementary material for: Anatomical-guided third-generation laser balloon ablation for the treatment of paroxysmal atrial fibrillation assessed by continuous rhythm monitoring: results from a multicentre prospective study
Source: Europace. 2024 Nov 7;26(11):euae263. doi: 10.1093/europace/euae263 (PMC11542219; doi:10.1093/europace/euae263)
Supplement: euae263_Supplementary_Data [file euae263_supplementary_data.docx]

**Supplementary Table 1.** Clinical and procedural characteristics of patients at different enrolling centers.

|  | **Centre 1** | **Centre 2** | **Centre 3** | **Centre 4** | **1 vs 2** | **1 vs 3** | **1 vs 4** | **2 vs 3** | **2 vs 4** | **3 vs 4** | **Global p-value** |
| --- | --- | --- | --- | --- | --- | --- | --- | --- | --- | --- | --- |
| **Age (Years)** | 57.59±10.87 | 60.20±10.17 | 65.30±11.52 | 59.55±11.09 | 0,152 | 0,001* | 0,477 | 0,017* | 0,808 | 0,055 | 0,004* |
| **Female, n (%)** | 17 (26) | 19 (27) | 18 (39) | 4 (18) | 1,000 | 0,155 | 0,571 | 0,222 | 0,574 | 0,103 | 1,000 |
| **BMI (kg/m2)** | 25.97±4.04 | 26.17±4.12 | 26.81±5.26 | 24.67±2.24 | 0,775 | 0,362 | 0,116 | 0,495 | 0,073 | 0,036* | 0,436 |
| **Heart failure, n (%)** | 3 (5) | 4 (6) | 2 (4) | 0 (0) | 1,000 | 1,000 | 0,568 | 1,000 | 0,569 | 1,000 | 1,000 |
| **CAD, n (%)** | 8 (12) | 3 (4) | 4 (9) | 2 (9) | 0,117 | 0,758 | 1,000 | 0,431 | 0,589 | 1,000 | 0,117 |
| **Previous TIA/stroke, n (%)** | 1 (2) | 4 (6) | 3 (7) | 0 (0) | 0,368 | 0,305 | 1,000 | 1,000 | 0,569 | 0,546 | 0,368 |
| **Hypertension, n (%)** | 36 (55) | 41 (58) | 30 (65) | 10 (46) | 0,863 | 0,332 | 0,466 | 0,445 | 0,337 | 0,187 | 0,863 |
| **Dyslipidemia, n (%)** | 34 (52) | 24 (34) | 24 (52) | 10 (46) | 0,037* | 1,000 | 0,628 | 0,056 | 0,326 | 0,796 | 0,037* |
| **COPD, n (%)** | 1 (2) | 2 (3) | 0 (0) | 0 (0) | 1,000 | 1,000 | 1,000 | 0,519 | 1,000 | 1,000 | 1,000 |
| **Diabetes, n (%)** | 4 (6) | 6 (8) | 6 (13) | 1 (4) | 0,747 | 0,314 | 1,000 | 0,536 | 1,000 | 0,414 | 0,747 |
| **OSAS, n (%)** | 6 (9) | 0 (0) | 4 (9) | 2 (9) | 1,000 | 1,000 | 1,000 | 1,000 | 1,000 | 1,000 | 1,000 |
| **LVEF (%)** | 57.35±4.74 | 56.04±15.98 | 53.59±19.78 | 59.86±4.92 | 1,000 | 0,800 | 0,751 | 0,803 | 0,754 | 1,000 | 0,297 |
| **LA volume/BSA (mL/mq)** | 30.85±7.72 | 31.09±7.23 | 31.41±6.83 | 33.60±6.59 | 0,853 | 0,688 | 0,114 | 0,809 | 0,136 | 0,213 | 0,474 |
| **Total procedure time (min)** | 42.52±12.12 | 53.73±15.40 | 58.52±16.12 | 63.41±13.20 | <0,001* | <0,001* | <0,001* | 0,114 | 0,006* | 0,191 | <0,001* |
| **Fluoroscopy time (min)** | 4.60±2.02 | 5.20±2.54 | 5.88±2.47 | 6.70±3.16 | 0,124 | 0,005* | 0,007* | 0,159 | 0,051 | 0,287 | 0,002* |
| **Pericardial effusion, n (%)** | 1 (2) | 1 (1) | 0 (0) | 0 (0) | 1,000 | 1,000 | 1,000 | 1,000 | 1,000 | 1,000 | 1,000 |
| **Persistent Phrenic nerve palsy, n (%)** | 0 (0) | 0 (0) | 3 (7) | 0 (0) | 1,000 | 0,068 | 1,000 | 0,058 | 1,000 | 0,546 | 1,000 |
| **Vascular access complications, n (%)** | 1 (2) | 1 (1) | 0 (0) | 0 (0) | 1,000 | 1,000 | 1,000 | 1,000 | 1,000 | 1,000 | 1,000 |
| **Balloon rupture, n (%)** | 0 (0) | 0 (0) | 2 (4) | 0 (0) | 1,000 | 0,170 | 1,000 | 0,153 | 1,000 | 1,000 | 1,000 |
| **ATs recurrences during the blanking period, n (%)** | 13 (20) | 15 (21) | 11 (24) | 4 (18) | 1,000 | 0,646 | 1,000 | 0,821 | 1,000 | 0,758 | 1,000 |
| **Freedom from any ATs recurrence, n (%)** |  |  |  |  |  |  |  |  |  |  |  |
| **12 months** | 56 (86.2%) | 60 (84.5%) | 39 (84.8%) | 18 (81.8%) | 0,977 | 1,000 | 0,883 | 1,000 | 1,000 | 1,000 | 0,320 |
| **24 months** | 53 (81.5%) | 57 (80.3%) | 36 (78.3%) | 18 (81.8%) | 1,000 | 0,853 | 1,000 | 0,976 | 1,000 | 0,985 | 0,400 |
| **36 months** | 51 (78.5%) | 54 (76.1%) | 34 (73.9%) | 16 (72.7%) | 0,897 | 0,741 | 0,795 | 0,966 | 0,973 | 1,000 | 0,450 |
| **ATs recurrence with burden ≥5%** | 3 (5) | 1 (1) | 3 (7) | 4 (18) | 0,348 | 0,691 | 0,065 | 0,298 | 0,011* | 0,202 | 0,348 |

Data are reported as mean±standard deviation or number (percentage) in case of binary variables.

Abbreviations: BMI = body mass index; CAD = coronary artery disease; TIA = transient ischemic attack; COPD = chronic obstructive pulmonary disease; OSAS = obstructive sleep apnea syndrome; LVEF = left ventricle ejection fraction; LA = left atrium; BSA = body surface area; ATs = atrial tachyarrhythmias

Significant values (p<0.05) are marked with an asterisk (*)

**Supplementary Table 2.** Clinical and procedural characteristics of patients undergoing third-generation laser balloon ablation using general anesthesia (GA) or deep sedation (DS).

|  | **General anesthesia (n=110)** | **Deep sedation (n=94)** | **p-value** |
| --- | --- | --- | --- |
| **Age (Years)** | 58.9±11.4 | 61.3±10.2 | 0.117 |
| **Male, n (%)** | 85 (77) | 61 (65) | 0.075 |
| **BMI (kg/m2)** | 25.8±3.8 | 26.6±4.8 | 0.181 |
| **Heart failure, n (%)** | 4 (4) | 5 (5) | 0.736 |
| **CAD, n (%)** | 8 (7) | 9 (10) | 0.740 |
| **Previous TIA/stroke, n (%)** | 3 (3) | 5 (5) | 0.475 |
| **Hypertension, n (%)** | 55 (50) | 62 (66) | 0.035* |
| **Dyslipidemia, n (%)** | 52 (47) | 40 (43) | 0.583 |
| **COPD, n (%)** | 1 (1) | 2 (2) | 0.596 |
| **Diabetes, n (%)** | 7 (6) | 10 (11) | 0.401 |
| **OSAS, n (%)** | 5 (5) | 7 (7) | 0.566 |
| **LVEF (%)** | 59.2±5.0 | 59.3±5.0 | 0.927 |
| **LA volume/BSA (mL/mq)** | 30.9±7.6 | 31.9±6.8 | 0.337 |
| **Total procedure time (min)** | 50.1±15.3 | 54.9±16.8 | 0.034* |
| **Fluoroscopy time (min)** | 5.0±2.7 | 5.7±2.3 | 0.049* |
| **Pericardial effusion, n (%)** | 2 (2) | 0 (0) | 0.501 |
| **Persistent Phrenic nerve palsy, n (%)** | 1 (1) | 2 (2) | 0.596 |
| **Vascular access complications, n (%)** | 0 (0) | 2 (2) | 0.212 |
| **Balloon rupture, n (%)** | 0 (0) | 2(2) | 0.212 |
| **ATs recurrences during the blanking period, n (%)** | 12 (11) | 12 (13) | 0.854 |
| **Freedom from any ATs recurrence, n (%)** |  |  |  |
| **12 months** | 95 (86) | 78 (83) | 0.641 |
| **24 months** | 90 (82) | 74 (79) | 0.714 |
| **36 months** | 84 (76) | 71 (76) | 0.822 |

Data are reported as mean±standard deviation or number (percentage) in case of binary variables.

Abbreviations: BMI = body mass index; CAD = coronary artery disease; TIA = transient ischemic attack; COPD = chronic obstructive pulmonary disease; OSAS = obstructive sleep apnea syndrome; LVEF = left ventricle ejection fraction; LA = left atrium; BSA = body surface area; ATs = atrial tachyarrhythmias

Significant values (p<0.05) are marked with an asterisk (*)
